# Lessons of Hope and Resilience: A Co-Produced Qualitative Study of the Experiences of Youth Living with Psychosis During the COVID-19 Pandemic in Nigeria

**DOI:** 10.1007/s10597-023-01128-8

**Published:** 2023-06-12

**Authors:** Tolulope Bella-Awusah, Haleem Abdurahman, Olubukola Omobowale, Olayinka Aturu, Adeola Afolayan, Olusegun Ogunmola, Bisola Fasoranti, Mayowa Olusanmi, Rita Tamambang, Olayinka Bamidele, Grace Ryan, Thomas Shakespeare, Julian Eaton, Olayinka Omigbodun

**Affiliations:** 1 Centre for Child and Adolescent Mental Health, College of Medicine, University of Ibadan, Ibadan, Nigeria; 2 Department of Child and Adolescent Psychiatry, University College Hospital, Ibadan, Nigeria; 3 Department of Psychiatry, College of Medicine, University of Ibadan, Ibadan, Nigeria; 4 Rehabilitative and Social Medicine Unit, Department of Community Medicine, College of Medicine, University of Ibadan, Ibadan, Nigeria; 5 Centre for Global Mental Health, London School of Hygiene and Tropical Medicine, London, UK; 6 CBM Global Disability and Inclusion, Laudenbach, Germany

**Keywords:** Psychosis, Co-Production, Youth, COVID–19, Pandemic, Nigeria

## Abstract

Much of the emerging evidence on the impact of COVID-19 on people with psychosocial disabilities comes from high-income countries. This study sought to explore the perceptions and experiences of youths living with psychosis during the COVID-19 pandemic in Nigeria. Using a co-produced research process, a facility-based study was conducted among youth with confirmed diagnosis of a psychotic disorder. In-depth interviews were conducted with 20 participants. Data was transcribed, double-coded and analysed with Atlas.ti using a thematic analysis approach. We found that participants were aware of good evidence-based information on the nature of the disease and the pandemic. Many of them described worsening mental health and disruptions to daily routines. Opportunities for deepening family relationships, skill building, helping others, and extended time for previously neglected self-development activities were described. This study benefitted from co-production with persons with lived experience, which could be harnessed for future research on psychosis.

## Introduction

In March 2020, the World Health Organization (WHO) declared the novel coronavirus COVID-19 outbreak a public health emergency of international concern (WHO, 2020a). Subsequently, governments all over the world began to adopt public health measures to contain the spread of the coronavirus. This resulted in lockdowns, physical distancing, and restrictions to public gatherings. Unfortunately, these measures were often detrimental to the wellbeing of the poor and vulnerable, particularly those living in low-and-middle-income-countries (LMIC) (Anglin et al., 2020). In countries like Nigeria, where there is no social protection, and a large proportion of the population live in poverty and survive on daily subsistence income with minimal or no savings, a situation of extreme poverty and even starvation may result if people are unable to pursue their livelihood activities. People confined to their homes also lacked opportunities to socialize, resulting in isolation, and increased rates of domestic violence. A lack of a balanced diet, exercise and access to healthcare services resulting in worsening physical and mental health (Brown et al., 2020; Kola et al., 2021) were also described. People with psychosocial disabilities are one of the most vulnerable groups worldwide, and preliminary reports from high income countries (HICs) suggest that people with psychosocial disabilities may be particularly vulnerable in the COVID-19 pandemic (WHO, 2020b).

Psychotic conditions are associated with the most severely disabling forms of psychosocial disabilities (Whiteford et al., 2015). People with psychosis often experience even greater stigma and discrimination, violence and abuse; restrictions in the exercise of their civil and political rights; and exclusion from social and economic opportunities than people with other forms of psychosocial disability. This greatly impairs their full participation in society (WHO, 2018). Increased levels of anxiety and fear, with uncertainty about the future are evident across the population, but may be further raised for those with a pre-existing psychosocial disability, increasing the negative impact they experience from the pandemic (Brown et al., 2020). Youth with psychosis are likely to be disproportionately affected by the psychosocial effects of COVID-19 (Omigbodun & Abdulmalik, 2020). They are more likely than their peers to drop out of school, and are more likely to be unemployed. The unemployment rate among youth living with psychosis is estimated to be as high as 70-80% (WHO, 2018). The social isolation resulting from the pandemic, the changes to the delivery of therapeutic services, and almost complete loss of all structured activities (school, work and training), and peer support networks put persons with psychosis at a higher risk of disadvantage (Kola et al., 2021; WHO, 2010). The responses to COVID-19 may also inadvertently exclude them, particularly as regards prevention and treatment activities. For example, information about COVID-19 may be communicated in formats that are not easily accessible to them, and social distancing may be difficult or impossible for those who require support from care givers.

Nigeria confirmed its COVID-19 index case in February 2020 and different degrees of restriction were imposed across different parts of the country ranging from partial to total lockdowns. These resulted in significant reductions in movement as well as economic and social activities across the country. These lockdowns led to devastating social and economic crises given the predominantly informal sector base of the Nigerian economy (Kola et al., 2021; Oyetunji et al., 2021). The health sector in turn shifted its focus to the care of persons infected with COVID-19 and the control of the pandemic, and there was a sudden shift of what was considered non-essential healthcare to virtual access without adequate preparation, leaving persons with persisting disorders, including serious mental illnesses, largely without much-needed care and support (Odubanjo, 2021). The important non-formal networks of religious and social gatherings were shut down and many had nowhere to turn to for support. A Nigerian qualitative study carried out among adults between the ages of 18 and 62 reported high levels of depressive and anxious feelings during the pandemic (Oguechi et al. 2021). The study also showed that coping mechanisms such as relaxation, engaging in pleasurable activities, hope and alcohol consumption were used by participants. A similar study in Northern Nigeria (Armiya’u et al., 2022) found that social support, engaging in pleasurable activities, hobbies, and spirituality were beneficial to the mental well – being of young people during the pandemic, while distressing feelings, the Covid − 19 restrictions, isolation and financial problems were detrimental to mental well-being.

In spite of the heightened vulnerabilities of youth living with psychosis in LMICs during the COVID-19 pandemic, most of the current evidence on the impact of the pandemic on this group is from high income countries. Little is currently known about the experiences of youth with psychosis in LMICs, although there may be similarities to the experience of young people with psychosis in more developed countries. Previous studies in the Nigerian and similar contexts have focused largely on common mental conditions such as anxiety, depression and stress-related issues among the general population, and frontline workers (Chakraborty et al., 2020; Oyadiran et al. 2020; Okediran et al. 2020; Olateju et al., 2022). It is likely that persons living with severe mental illnesses such as psychosis would be more vulnerable not only to the psychological impact of the pandemic, but also to the social and economic disruptions occasioned by the COVID-19 pandemic and the associated public health response (Brown et al., 2020). This study therefore set out to explore the perceptions, perspectives, and experiences of youth living with psychosis during the COVID-19 pandemic in Nigeria.

## Methods

### Study Design and Setting

This was a cross-sectional qualitative study conducted at the University College Hospital, a tertiary teaching public health facility located in Ibadan, Southwest Nigeria with specialised services for children and youths with mental health conditions. Ibadan is the third largest city in Nigeria with a population of about 6 million people made up of 5 urban and 6 semi-urban districts, and predominantly inhabited by the Yoruba tribal group. The main economic activities in Ibadan include agriculture, trade, public service employment, industry and education. The mental health service for children and youth sees patients up to the age of 25 years and comprises of outpatient clinics, an inpatient unit, consultation-liaison to other hospital- based units, and outreach services to community-based institutions such as mainstream and special schools, the juvenile justice system and the Comprehensive Health Service in the University of Ibadan. The service for children and youth was established in 2000 (Kang et al., 2021).

### Participants

Participants were eligible to be part of the study if they had a lived experience of a psychotic condition such as Schizophrenia, Bipolar disorder or schizoaffective disorder, and their age ranged from 18 to 25 years. Diagnoses were determined using the ICD − 10 diagnostic criteria and confirmed through patient records.

### Research Ethics

Ethical Approval to conduct the study was obtained from the University of Ibadan/University College Hospital Ethical Review Board with approval number UI/EC/20/0336. Written informed consent was obtained from each participant before data collection and all identifying information was removed from transcripts before data analysis. Participants received a token of N1,500 ($3) to offset their transport fees to the facility.

### Patient and Public Involvement

This study was based on the principles of co-production. Three researchers with lived experience of a psychotic illness (peer researchers) were involved in the conceptualization of the research idea, research protocol adaptation of the key informant guide, training on interviewing skills, recruitment of participants, transcription of data, coding of transcripts, thematic analysis, interpretation of the data, and manuscript write up.

### Recruitment and Data Collection

Before the commencement of data collection, team members underwent a 2-day training to develop and pilot the study instrument, as well as improve interviewing skills. Subsequently, 5 team members (2 males and 3 females) conducted the interviews. All interviewers were members of the research team of the SUpport, Comprehensive Care and Empowerment for people with Psychosocial Disabilities in Africa (SUCCEED) project. Interviews were conducted using a key informant interview guide co-produced by the Nigerian SUCCEED team in collaboration with the London School of Hygiene and Tropical Medicine (LHSTM) team (see appendix). The guide explored participants’ understanding of, and experiences during the COVID − 19 pandemic, including impact on their daily life, work, school and social support. A convenience sampling method was used to recruit eligible participants from the Child and Youth Mental Health Service. All eligible participants found in the service records were contacted by telephone, and provided with information on the study. Service users who were unable to give informed consent were excluded from the study. Data was collected over a period of 8 months, (September 2020 – May 2021) until data saturation was reached. All interviews were conducted face-to-face at the outpatient clinic in accordance with COVID-19 safety regulations; audio-recorded, and lasted between 40 and 90 min. Interviews were conducted in English or Yoruba (the local language), depending on participants’ preferences. Participants also filled out a short socio-demographic form to determine their age, gender, religion, and level of education.

### Data Analysis

All interviews were transcribed verbatim. Four members of the team, including the peer researchers, did a quality check to ensure fidelity. Coding was an iterative process following a thematic analysis approach. Three members of the research team (TB-A, OO^4^ and AA) initially read through all the transcripts to familiarize themselves with the data and independently coded five of the transcripts inductively using Atlas.ti version 7 qualitative research software. Researchers refined codes and developed a coding framework for the remaining transcripts (Braun & Clark, 2006). This framework was used to independently code the remaining 15 transcripts. Researchers met again to discuss the new codes generated, and identify emerging themes. In a final meeting, as part of integrating experiential knowledge into the interpretive process, three peer researchers (two of whom had independently coded two transcripts each manually) contributed to the finalisation of themes and subthemes. The approach to data collection, coding and analysis was guided by the Consolidated Criteria for Reporting Qualitative research (COREQ) (Tong et al., 2007). To ensure the validity and rigour of the findings (Morse et al. 2015), the researchers used a number of established approaches including development of a coding system, peer review of themes, detailed description of the context where data was collected, and supporting quotes.

## Results

### Participant Characteristics

A total of 20 participants (11 males, and 9 females) participated in the study. Their mean age was 20.2 years (SD = 1.9) and the majority (85%) were university students (see Table 1).

**Table 1:** Socio-demographic information of Participants (N = 20)

Description	Frequency	Percentage (%)
**Gender**		
Men	11	55 0
Women	9	35.0
**Age**		
Age in years (Mean, SD)	20.2 (1.9)	
Age range (years)	18–25	
**Religion**		
Islam	3	15.0
Christianity	17	85.0
**Living with**		
Parents	18	90.0
Others*	2	10.0
**Current Educational Level**		
Secondary	3	15.0
Tertiary	17	85.0
**Diagnosis**		
Bipolar Disorder	7	35.0
Schizophrenia	10	50.0
Schizoaffective disorder	2	10.0
Unspecified Psychotic Disorder	1	5.0

*Others include living with a relative or living alone

### Key Themes

The findings are presented under six themes: general awareness of the pandemic, initial thoughts and reactions, mental health and services impact, life impact, coping with the pandemic, lessons learned and recommendations for the future (see Table 2).

**Table 2:** Themes and sub- themes derived from the study

S/N	Theme	Subtheme
1.	General Awareness	Varying levels of knowledge on the virus, its cause, mode of transmission, public health measures
		Preventive measures of the virus
		Less deadly in Africa
2.	Initial reactions	Fear and Anxiety
		Indifference
		Relief
3.	Impact on mental Health/ Services	Varied impact on mental health
		Reduced access to in – person services
		Reduced access to medications
		Exploration of other mental health care options
4.	Impact on life circumstances	Financial hardship
		Insecurity of food and other daily needs
		Disruptions to daily routines and religious activities
		More online presence
		Challenges with adherence to public health measures
		New opportunities and skills
		More time for other important activities
5.	Coping	Recreational activities
		Seeking inspiration
		Connecting with others
		Comfort eating
		Cultivating a positive mindset
6.	Lessons learnt/ recommendations	Government controls our lives
		Always be prepared
		Need for stigma reduction
		Equity in mental health care
		Family empowerment

### General Awareness of the Pandemic

All participants had a general awareness of the pandemic, and that it originated from China. However, knowledge about the causes, and public health measures for COVID-19 varied. With regards to aetiology, most participants mentioned a virus caused it. A few participants were unsure as to the cause of the pandemic, while others felt the pandemic was a form of biological warfare, an attempt to reduce the population of the world, a failed laboratory experiment, or a punishment for sin.

*I’m thinking that the world government wants to use it to reduce the population of the world* […] *because there are limited resources that the earth has to provide for the people that are alive, so they found a way to create the COVID-19 virus to start killing people.* (20-year-old man).

[…] *according to what I’ve heard, like they said maybe God is angry and He wants to teach us some lessons so He sent down a virus to the world such that none is going to escape* […] *ok now* [it] *is like ‘I want to punish you for your sins, like sins you’ve committed,’ so I think like what they’ve said is kind of true, that God is angry and He’s the only one that can take away the virus.* (19-year-old woman).

Participants also had various different explanations for the mode of transmission of the disease, such as blood transfusions and breastfeeding; others described the standard scientific explanation, i.e., respiratory droplets:

[…] *it can stay in the air for a long period of time, so the mode of transmission is easy to breathe in via air droplets. So, when someone beside you coughs or something or sneezes, you can easily get infected from the virus.* (22-year-old woman).

All participants knew about the various public health measures put in place to curtail the spread of the virus, such as use of face masks, hand washing and sanitizers, social distancing, and government mandated lockdowns. Participants had mixed beliefs about the effectiveness of these measures:

*But if you try to follow the protocols given by the Nigerian Centre for Disease Control, this kind of thing…at least you are 50% safe from this disease.* (18-year-old man);

[…] in Nigeria, well people were not obeying [the public health measures]. I know that for sure because you see people at the market… Everywhere […]. (20-year-old woman).

Some participants mentioned alternative preventive measures for the virus such as herbs, vitamins, special prayers, and religious affiliations:

*Ah. I will not lie to you, it’s prayer […] Because, God really answers my prayer. The road where other people are passing, that’s where I’m passing, and those people contract the diseases, they contract COVID-19. But not me […].* (19-year-old man).

*No, I don’t think I will contract the disease, because God is on my side […] God is on my side [laughs] and Jesus’ blood is on my head. (20-year-old woman).*

Many mentioned that thankfully the first and second waves of the pandemic had not been as deadly in Nigeria as in Asia, and parts of the Western world:

*Nigeria is not really one of those countries that is affected by the pandemic when it comes to statistics. We are not one of the top ten countries.* (24-year-old woman).

Participants felt Africa had been spared of the most devastating effects of the pandemic and would remain so.

### Initial Thoughts and Reactions

The majority of participants mentioned fear, anxiety, and disbelief as their initial reactions to the pandemic. Many were scared of contracting the virus, dying or losing their loved ones:

*The greatest fear was contracting the virus, that was just it. I didn’t want to get infected.* (22-year-old woman).

*Losing a loved one, that was my greatest fear, because I can’t afford to lose anyone. I’ve been hearing of many people dying. People have been losing their dads, losing their mothers, losing their brothers […]. I was always, I was constantly saying, ‘God please let me not lose anyone.* (22-year-old man).

Other fears were of the unknown, uncertainty created by the pandemic, and the possibility that the pandemic would lead to the end the world:

*Ok, I was afraid that the world is coming to an end, that we would die off at some point.* (21-year-old woman).

A few participants were afraid that things would get to a point when they would be unable to access basic needs like food.

On the other hand, the initial reactions of some participants would be described as minimal or even positive

*[…] at first, I was actually indifferent because we just finished school, we were having our break and they told us there was COVID-19 outbreak, we were actually on holiday already so there’s no difference at all.* (19-year-old man)..

Alternatively, the pandemic could be seen as a relief from pressure to perform well in coursework or exams:

*When we were in school, they stressed us even while writing our project. So, when the pandemic started, we were grateful to God.* (22-year-old man).

### Impact on Mental Health and Services

Participants described a variety of emotions as the pandemic progressed. Some found their mental health condition improved or at least they reported that they remained stable, while for others, they reported that it worsened considerably. For example, one participant reported better mental well-being, which he attributed to increased family support: *[It’s] funny, but I got better, because I saw better signs […] staying at home with my family made me better because most of the cause of my problems were loneliness and stress*, (19-year-old man). Others reported no adverse impacts: *My mental health condition, I guess it has been better for a long while now, so I don’t think it affected me negatively*, (19-year-old woman). Meanwhile, negative emotions described by participants included loneliness, boredom, and lack of motivation *(It was a boring period, [20-year-old woman])*, leading to loss of productivity:

*I wasn’t so productive. Because I felt like I could do better. There’s this blog I run and I should have written more poems and put [them] there but I don’t know, I was just […] It was a lazy period for me* [*laughs*]. (22-year-old man).

Several participants reported worsening of their mental health symptoms during the pandemic. These symptoms ranged from anxiety to psychotic and pseudo-neurological symptoms, to suicidal thoughts.

*I was suicidal then. Funny enough during the early hours of the day, I was not suicidal, it usually happened in the night like 7–8 pm. I would feel like I can’t take it any longer [……], I was feeling fed up.* (24-year-old woman).

One participant was in such distress that she said: *During the pandemic, my greatest fear had nothing to do with COVID-19. My greatest fear was that one day I would self-destruct*. (19-year-old woman).

Participants described reduced access to in-person mental health services as a result of travel and movement restrictions as well as reluctance to visit the hospital for fear of contracting the virus: *During the lockdown there was restriction in traveling, yes and again hospitals were not really the safe place to be*, (24-year-old woman). Some participants had remote access to their mental health care providers via mobile phones, but all those who had this access reported that it was less satisfactory than regular in-person access:

*We had to communicate by phone most of the time; had to talk to the doctor on phone. It’s not really easy, I prefer talking to them one-on-one because you get to remember things as they are asking you those questions, you get to remember more things and all. It’s not just the same as talking to them on phone.* (22-year-old woman).

Access to medications was also reduced due to drugs scarcity resulting from disruptions to the supply chain as well as cost increases:

*When they write the medication for us, and we go to the pharmacy, the price that they will charge us will be very much. So, we have to buy half, we will have to buy for two weeks. After that is finished, we will now go and re-buy it again.* (18-year-old man).

Participants also found themselves exploring other mental health care options through their primary health care providers, as well as online alternatives: *So, I was on this WhatsApp group which runs once a month. The moderator has a lot of knowledge about mental health and he sends us voice notes. So, I listen to the voice notes and inculcate the advice into my life…. so that is how I found free therapy* (21-year-old man); *I have a family doctor […] So I call him if I feel anything before, I call my psychiatrist […] like the time when I was having trouble sleeping, I quickly called him. He gave me diazepam. I used it for about two days and I started sleeping better […].* (21-year-old man).

### Impact on Life Circumstances

Participants described a decline in the financial resources available to their families as a result of unpaid salaries and businesses being locked down: *[…] my dad’s business was not really going smoothly. He was always complaining about business like things were bad, people were no more patronizing [no longer patronizing the business] and things were just [……]. It really affected the business during that period.* (22-year-old man).

Participants also described problems getting food, because of challenges transporting goods, closed markets, and resultant hikes in food prices: *[…] they kind of reduced the market days so sometimes for 3 days straight, there won’t be any food available [….], so it affected us getting food.* (19-year-old man).

Most participants described disruptions to their daily routines particularly in the areas of school, work, leisure, and religious activities. Most of our participants (18/20) were able to move these activities online through their mobile phones:

*We were not able to go to school for about six months or something. Now we do online lectures, but it’s not like the physical one. Some people do not grab online classes. Like me, I do not prefer online classes. I like physical* [classes more] *than online* [classes]. (18-year-old man).

However not everyone could perform these activities online and one participant complained of not being able to attend church: *I’m a Catholic, not going to church on Palm Sunday was very horrific. It was the day of Easter celebration and I was at home, my mum had to conduct the service in our house, it was odd.* (21-year-old female). Some of those who were able to go online complained of insufficient funds to buy data.

Participants described various challenges they had with some public health measures such as the use of face masks which made breathing difficult, and social distancing which was inconvenient and often not remembered: *“But that nose mask makes it difficult for people to breathe. You know we need to breathe in air. Sometimes, it prevents one from breathing properly.* (22-year-old man).

*Hm, the social distancing was quite difficult. [Cos] I’m this kind of person that likes being free with my friends. So, when I see my friends, the next thing you do as guys is shake hands or we laugh and then we hit each other or stuff like that. But [chuckles] because of the Corona virus we couldn’t do that anymore. […] most times you would have already shaken your friend before you realize that you are not supposed to […] it was just somehow for me […].* (22-year-old man).

Participants also mentioned a number of positive effects of the pandemic on their lives.

Participants were able to learn new skills such as baking, bee keeping, graphic designing, and start new jobs and businesses with the skills learnt: *Yes, during the pandemic, I started learning about bee keeping. Hmm, it’s generating a little income. When we harvest and we sell, we get some money*, (22-year-old man). Others volunteered with local organisations to bring succor to others during the pandemic: *I’m a volunteer at an NGO and they needed funds, so I helped to solicit for donations, we also went for an outreach, and we distributed food* (19-year-old woman).

Participants also mentioned having more time for other important activities such as resting, sleeping, catching up on schoolwork, personal reflection, spiritual growth, and spending quality time with family members:

*On a normal day before the corona, I don’t have any time to sleep. The only time I had to sleep is late in the night because I had to read overnight. But corona has blessed me with enough time to sleep.* (21-year-old man).

*Like actually in my own family, we have enough time, we have more, more time for ourselves like gisting, [chatting] praying together, eating together and doing many things together.* (20-year-old woman).

Many of the participants described improved support from family members as a result of the extended time spent with family members: *The people that I have around me are people that will go extra length to support me, they get my medications, cook for me, clean the house… yeah, they are really supportive.* (18-year-old man).

### Coping with the Pandemic

Participants used several types of recreational activities to cope. These included watching movies and comedies and listening to music: *I listen to Nigerian music by artists like Burna boy and Whizkid. It helps, it boosts my mood*, (19-year-old man). Others read books, played games, spent time with family members and exercised: *I also read books. I read basically all the novels in the house because there was no electricity to watch television… so the novels keep me busy until I dose off.* (21-year-old woman).

*I have a baby sister with whom I play a lot with and she is very funny. We play kung fu, she fights, she tries to carry me and all. Then my mum is a very jovial person, she really plays, she’s very playful. So, we all try to play together.* (22-year-old woman).

Several participants also sought inspiration through reading religious texts and watching or listening to motivational stories online:

*And I was, what else, I think I was on social media, twitter, I was tweeting. I was very active on social media during this period, so it helped me stay positive, and I saw stories of other people. How people go through various life challenges and how they were able to cope with it and emerge strong. So, it kept me going.* (22-year-old man).

Participants described staying connected with others especially family and friends online as an important coping mechanism for them: *“Well, the good thing is that I was able to keep in touch with family members, especially my brothers and sisters, so keeping touch with them was like a good feeling for me. We talked on WhatsApp, we made video calls”*. (18-year-old man). A few participants who had difficulties going online were able to physically keep in touch with friends who lived close by. Several participants sought emotional support from others as a way to cope during the pandemic. Preferred go-to persons to discuss problems were family members, friends and therapists:

*After I’m done thinking and my head is very heavy, I call either mum or my uncle. And fortunately, one of my uncle’s wife is a therapist. So, we could talk and she would talk to me that, don’t worry, you just have to maintain it […] So, she will motivate me, tell me this and that, and I’ll be like, Okay […].* (21-year-old man).

A few participants mentioned eating more and eating junk food whenever they felt stressed: *I grew a little bit bigger because I was eating a lot of ice cream and cake [….]* (22-year-old woman). Some maintained a positive mindset to enable them to cope. For example, one participant kept telling herself that the pandemic would eventually pass and life would go on:

*Well, I know that I’m not the only one in this. I know that we will resume school and I will come back stronger. I know that it* [the pandemic] *will go away. That’s my thought that it will go away. I will not have to see this kind of pandemic again. That has been my consolation.* (18-year-old man).

### Lessons Learnt and Recommendations for the Future

Summarizing the main lessons that participants reported from this period, two key messages emerged: first, that the government has a lot of control over people’s lives; and second, to be prepared for anything. A few participants felt the government could change the lives of citizens at will, as exemplified by the lockdowns imposed, and citizens were helpless to do anything about it, as described by this young woman:

*The fact is that nobody is independent in Nigeria, not even the churches. I just felt that the government is the overlord, because you know, when everything started, the churches were locked down, there’s nothing they could do. So, we that are religious, that used to rush to our churches as our last resort, we started training our minds that, “Oh there is something bigger than this; after God it is the government, so something like that.* (21-year-old woman).

The other important lesson was to always be prepared for whatever life throws at one: *The thing is don’t always expect things to go as you’ve predicted [….] So, it is better for you to just have this kind of mindset of, ‘It may work, and it might not work.’ So, don’t get depressed if it doesn’t work.* (22-year-old woman).

Recommendations to families, government and civil society for supporting persons with psychosocial disabilities post-COVID centered on stigma reduction, equity in mental health care, and empowerment of families. Participants wanted families and society at large to stop stigmatizing people living with mental health conditions and the government to provide public education in this respect:

*Well, what communities can do is that they should sensitize the communities too about the illness, they should tell them about the symptoms of the illness in case any person is having those symptoms, so they will know the right step to take. So that they won’t go and tie them in one place and start beating them because they are mad or something.* (20-year-old woman).

Participants also spoke about a variety of changes they wanted to see in mental health care. Paramount was the need for more facilities and personnel, subsidized services and medications, early access to services, and patient-centered care. Those who had virtual appointments through telephone calls and texts wanted more sophisticated digital methods such as Skype or video conferencing to be introduced. A few participants mentioned inclusive mental health research (co-production), which would allow for open discussions between service users and mental health care providers.

*I even want a thing where I can actually sit down with other people that have mental health conditions, and then we also have a board of doctors, and each of us will explain how it happened and what it took from us, like how it really affected our lives in various ways. They too they will have to understand that we too, that they are even taking care of, are going through hell*. (21-year-old man).

Participants wanted families to be educated on mental health so they could better support people living with mental health conditions. They also spoke of the need for the economic empowerment of families to enable them to better cater for the needs of family members living with mental health conditions.

*You know most of the time, the family members, they don’t understand what you are going through. So, it’s the doctors that explains* [sic] *very well and in cases when probably you are not lucky and your doctor don’t* [sic] *get to explain to you, the family members may misinterpret your behaviour, they feel probably you are just being proud, you don’t want to work at home when you are depressed. You can imagine! So, for family members, I feel they should be well educated.* (22-year-old woman).

## Discussion

This co-produced qualitative study explored the perceptions and experiences of youth living with psychosis during the COVID-19 pandemic in Nigeria. There are aspects about the participants in this co-produced study that are important to note in interpreting the findings. The majority (85%) of the participants in this study were university students, they had access to a mobile phone and to data to have been recruited and they were also among the less than 20% of persons with mental health conditions who are able to access formal care (Wang et al., 2007). They were contacted from the attendance records of the Child and Youth Mental Health service in Nigeria’s foremost and first Teaching Hospital. In this regard it appears that the youth living with psychosis who were interviewed were mostly those who were functioning well enough to have gained entrance into a tertiary institution and also privileged to have been able to access the services of a psychiatrist and a multidisciplinary team at this hospital (Omigbodun, 2004; Kang et al., 2021). In addition, the Child and Youth service in this hospital has an outreach to the University Health Service and hence the large proportion who might have been picked up at the University Mental Health clinic and referred.

Study participants had a working knowledge of the nature and causes of the pandemic, as well as its origin and mode of transmission mixed with some alternative theories. A lot of religious content was deeply interwoven into our participants’ beliefs about causation, vulnerability to, and treatments for the COVID − 19 virus. This may be attributed to the African traditional view of reality which is one of interdependence between man, nature, and the spirit world (Omonzejele, 2008; White, 2015). Illness causation is often thought of as two-fold: the organic cause, and the metaphysical cause, and a popular metaphysical theory for illness in the African context is the anger of the gods, which was mentioned by several of our participants (Omigbodun et al., 2001). All participants were aware of at least one or two public health measures that had been put in place to curb the spread of the virus. Though many of our participants were aware of various alternative preventive health measures, the only alternative measure they resorted to was special prayers. This was borne out of the fear that the alternative biological agents could lead to drug interactions with their psychotropic medications, leading to greater distress.

Like others around the world, our participants experienced distressing emotions at the onset and throughout the progression of the pandemic (Branquinho et al. 2020; Scott et al., 2021; Rains et al., 2021). Distressing emotions such as fear were related to catching the virus, dying, losing loved ones, and the general uncertainty created by the pandemic. These feelings later evolved into feelings of loneliness, boredom and amotivation as the pandemic progressed. A UK study conducted mainly among older mental health service users and caregivers (mean age 42.61 years) identified similarly negative emotional reactions such as fear, boredom, and guilt (Simblett et al., 2021). Another study carried out among young people in Portugal (mean age 19 years) reported increases in feelings of anxiety and depression during the initial phase of the pandemic (Branquinho et al., 2020). A few of our participants, who were under pressure at college, experienced neutral or positive emotions, such as indifference or relief. Similar positive reactions of happiness and calm have been reported in other studies among adolescents and older people with and without pre-existing mental health problems (Branquinho et al., 2020; Scott et al., 2021; Simblett et al., 2021) which were attributed to fewer demands on participants’ time and a slower life pace.

The mental health impact of the pandemic varied among our study participants. Expectedly, some of them described a worsening of symptoms or remained stable. Rains et al. in their international review of experiences of mental health service users during the pandemic also found that the impact of the pandemic on the mental wellbeing of people with pre-existing mental health conditions across the world was varied and wide, with reported increases in anxiety, panic attacks, sleep problems and thoughts of self-harm, as well as increased comfort and confidence for others. Isolating polices of lockdowns and social distancing, loss of support from health and other services, and effects of increased social adversities, such as domestic abuse, family conflict, or loss of employment, were identified as underlying causes (Rains et al., 2021). Surprisingly some participants felt their mental health improved during the period of the pandemic. This may be attributed to reductions in overall stress through reduced school and work pressures, more time to sleep and relax, as well as increased family support described by several participants.

Surprisingly, none of our participants described instances of family conflict or domestic abuse during the pandemic. The reasons for this are not very clear, but it is possible that those from very stable, supportive homes were more likely to consent to participate in the study further buttressed by the fact that some of the participants were very happy for the additional family time together during the lockdown. Also, the issue of living in overcrowded home circumstances, which is quite prevalent among the urban poor where the entire family would live in one room and share amenities with several other families did not arise. This is an indication that the participants might have been a more privileged select group of youth living with psychosis. Many families in urban slums live in houses with up to 100 people sharing amenities such as bathroom and kitchen. These groups are more prone to domestic violence and other vices (Omigbodun & Abdulmalik, 2020). It is also possible that the lack of reporting of domestic abuse in this study is in line with general under – reporting of such incidents in the community (Gracia, 2004; Alhie, 2009).

With regard to services, in-person mental health services were closed down at the facility from which participants were recruited in June 2020 at the peak of the first wave of the COVID 19 pandemic. Subsequently, all services except emergency care were carried out remotely through phone calls and messaging. While mobile phones are widely used in this context, younger people still have less access and may depend on parents and guardians for mobile phone use leading to a lack of privacy and confidentiality. It appears that not all participants received information detailing the new options for remote care. This may account for participants’ responses related to access, i.e., movement restrictions and fears of getting infected if they visited the hospital.

For those who were able to access remote services, there were complaints that they missed the usual face-to-face interactions, which appeared to reduce their satisfaction with the therapeutic interaction. Other studies have noted similar experiences among service users, including more superficial therapeutic contacts, as well as the loss of a therapeutic safe space (Liberati et al., 2021; Rains et al., 2021). In addition to problems with accessing clinic services, our participants also had problems with accessing medications. Because of the restrictions in movement of people and goods, prices had been hiked, and many were panic-buying, leading to scarcity of many commodities, including medications. The inability to secure their usual medications may have contributed to the worsening of mental health symptoms for some of our participants. Other studies have also reported service users complaining of disruptions to supply of medications as well as disruption to the in-person contacts required to prescribe, monitor, and administer medications (Rains et al., 2021).

Participants described a range of negative impacts of the pandemic similar to those reported by other service users around the world (Gilliard et al. 2021; Simblett et al., 2021; Rains et al., 2021). However, participants also reported positive impacts such as the creation of new opportunities, more time for previously neglected activities, and enjoying more support from their loved ones. Though some participants reported losing motivation and passion during the pandemic, others harnessed opportunities for self-development, growth, and helping others. Coping methods were largely emotion focused either by the use of pleasurable activities to distract themselves, or by seeking support from others. These findings are in line with other recent studies around the world which have shown similar coping, resilience and resourcefulness displayed by people living in vulnerable situations during the pandemic (Gilliard et al. 2021; Rains et al., 2021; Simblett et al., 2021).

Going forward into a post-COVID world, participants desired greater equity in mental health services. They wanted improved availability of affordable and accessible mental health services within their communities. Patient-centered care was vital for them, and the need for more evidence-based practices which would include the wishes and opinions of service users. They wanted families, (who are the main source of support and social care for persons with psychosocial disabilities in this context), to be empowered and supported to be able to improve in carrying out this role. These desires are in line with the growing call for a rights-based approach to mental health, such that persons with mental disorders can fully participate in the planning, monitoring and evaluation of mental health services, systems and research. Communities should also be strengthened and empowered to be at the forefront of all activities promoting mental health and well-being for all (UN, 2017).

Overall, participants in this study appear to have coped unexpectedly well with the challenges presented by the pandemic, and this may be related to the factors highlighted earlier where most of them appeared to be from better-resourced families who were able to provide a great deal of psychosocial support during the initial phases of the pandemic. This may have helped to cushion the effects of the pandemic. Another reason might be that most of the participants were recruited during the first phase of the COVID-19 pandemic, which had lower mortality rates in Nigeria than the second and third waves, possibly as a result of the lockdowns put in place. Thirdly, families are the main source of social care in this environment, so our participants did not suffer the loss of access to social services in the same way that those in higher-income settings might.

### Strengths and Limitations of the Study

This is one of the first studies co-produced from a LMIC focusing on experiences of the COVID-19 pandemic among young people living with psychosis, who are often left behind in disaster response and in health and social policy more broadly (WHO, 2010). Previous studies in this context have focused mainly on the mental health and experiences of the wider population affected by COVID-19, as well as of some specific groups like frontline health workers who often work in these difficult situations (Erinoso et al., 2020). Co-production principles also enhanced this study with the experiences of peer researchers in drawing up the interview guide and interpreting the findings. However, there were some limitations to our study. First, participants were recruited from a tertiary mental health facility, which is mostly accessible to people of higher socio-economic status living in urban areas. Hence the views expressed in this study may not be representative of all young persons living with psychosis in this context, and are certainly not representative of the 3–5% of people with severely disabling mental health conditions in Nigeria many of whom are unable to access mental health care (Gureje et al., 2006). Participants in this study were also recruited via phone, and this could have introduced some bias into our selection process, as we have highlighted that phone access is often mediated by parents or other family members. We also excluded participants who could not give informed consent probably inadvertently leaving out the voices of the most vulnerable members of this group. “Future studies could sample young persons living with psychosis directly from the community, including urban and rural communities, for a more representative sample”.

## Conclusion

This study explored the understanding and experiences of young people living with psychosis during the COVID-19 pandemic in a LMIC like Nigeria. Participants had a good knowledge of the COVID-19 pandemic. They experienced a wide range of negative effects from the pandemic, but were also able to harness inner resources to cope. These findings are similar to those from higher income countries, though economic risk was increased. There is a need for ongoing collaborative research to understand the long-term effects of the pandemic on people with psychosocial disabilities. At present the needs of people with psychosocial disabilities are poorly served and the pandemic has raised the profile of mental health and wellbeing. Appropriate research, and capitalizing of this opportunity of increased attention will aid service planning to meet needs in a holistic way.
